# Stifle arthrodesis in a feline pelvic limb amputee

**DOI:** 10.1177/20551169231217837

**Published:** 2024-01-10

**Authors:** Wei Tze Goh, Jarrod Drew

**Affiliations:** 1 College of Veterinary Medicine, Murdoch University, Murdoch, WA, Australia; 2 Animal Referral Hospital, Sinnamon Park, QLD, Australia

**Keywords:** Stifle, arthrodesis, hindlimb, amputee, salvage

## Abstract

**Case summary:**

A domestic shorthair cat presented to the Animal Referral Hospital (Brisbane, Australia) after having the left pelvic limb incorrectly amputated. The cat was unable to ambulate on the remaining right pelvic limb due to a chronically subluxated stifle. A stifle arthrodesis was performed on the right pelvic limb to manage the injury. Follow-up radiographs performed 5 months postoperatively demonstrated stifle arthrodesis with no detectable complications. The owner reported that aside from some difficulties in toileting, the cat had a good quality of life and was capable of performing the majority of daily activities. Stifle arthrodesis in a feline pelvic limb amputee appears to be a viable treatment option. After a period of rehabilitation and adaptation, the cat in the case report has been able to lead a near-normal lifestyle.

**Relevance and novel information:**

To the authors’ knowledge, this is the first report of the outcome and complications associated with stifle arthrodesis in a feline pelvic limb amputee. This is also the first report of stifle arthrodesis in a cat using the bilateral plating technique.

## Introduction

The primary stabilising structures of the stifle joint consist of the cranial and caudal cruciate ligaments and the medial and lateral collateral ligaments.^1^ Luxation of the feline stifle joint typically occurs after a traumatic event that generates large extrinsic bending and torsional forces, subsequently damaging both the cranial and caudal cruciate ligaments and the medial and lateral collateral ligaments.^2–4^

Reported treatments for multiligamentous stifle injuries include ligament reconstruction or prosthesis, trans-articular external skeletal fixation, total knee replacement and stifle arthrodesis.^5–7^ Stifle joint arthrodesis is an alternative to amputation and is indicated in the case of stifle subluxation/luxation, failure in primary joint reconstruction and when total knee replacement is precluded due to clinical or financial grounds.^6–9^ A previous case report demonstrated good long-term outcomes after a stifle arthrodesis in two cats.^6^ However, we are unaware of any case reports that document the outcome of stifle arthrodesis in a feline pelvic limb amputee. This case report details the surgical technique, complications and long-term outcome after stifle arthrodesis in a feline pelvic limb amputee.

## Case description

An 11-year-old, male neutered, 6.4 kg domestic shorthair cat was presented to a primary care veterinarian for acute onset non-weightbearing lameness of the right pelvic limb after returning from outdoors. On physical examination, laxity of the stifle joint was noted. Radiographs revealed a cranial luxation of the right stifle. Amputation was elected due to financial constraints and was performed approximately 4 weeks after the initial diagnosis. However, the left pelvic limb was amputated instead of the right one, due to human error.

The cat was then referred to our hospital for further management. Closed reduction attempts under general anaesthesia were unsuccessful due to the chronicity of the injury. Consequently, a stifle arthrodesis was recommended.^8^

**Figure 1:**
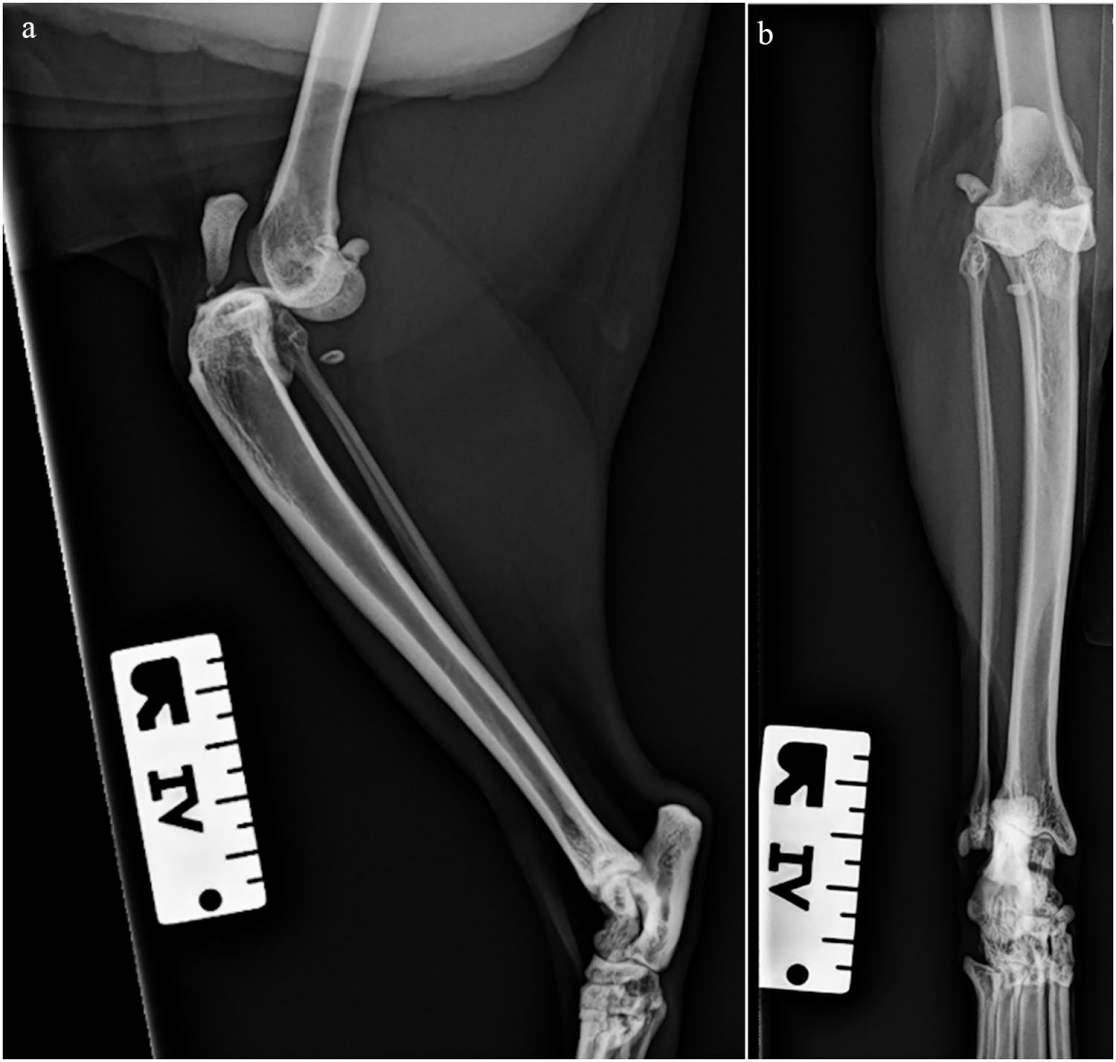
(a) Preoperative mediolateral radiograph of the right stifle. Marked cranial displacement of the tibia in relation to the femur was noted. (b) Craniocaudal radiograph of the right stifle. Proximal displacement of the tibia with mild overriding of the stifle joint was noted

Surgery was delayed for 10 days to allow management of an episode of idiopathic cystitis resulting in severe haematuria requiring blood transfusion. Once resolved, radiographs were obtained under general anaesthesia, which demonstrated stifle effusion and cranial luxation of the stifle (Figure 1). Stressed radiographs were not performed as the stifle had limited mobility.

A craniomedial approach to the right femur and tibia that extended from the femoral mid-diaphysis to the tibial mid-diaphysis was performed. A tibial tuberosity osteotomy was then performed with a sagittal saw to allow proximal retraction of the quadriceps muscle group to improve stifle exposure.^10^ A 1 mm K-wire was placed perpendicular to the long axis of the distal femur in the sagittal plane. A second 1 mm K-wire was then placed distal to the femoral K-wire, 30° from perpendicular in the sagittal plane, in a craniodistal to caudoproximal orientation. Another 1 mm K-wire was then placed perpendicular to the long axis of the proximal tibia in the sagittal plane. A 1 mm K-wire was then placed proximal to the tibial K-wire, 30° from perpendicular in the sagittal plane, in a cranioproximal to caudodistal orientation. The angled K-wires were used as ostectomy guides to achieve a stifle arthrodesis angle of 120°. A sagittal saw was then used to perform an ostectomy of the distal femur and proximal tibia parallel to the angled K-wires. The stifle was stabilised using two crossed 1.4 mm K-wires and the four 1 mm K-wires were then removed. A 15-hole 2.7 mm String-of-Pearls (SOP) (Orthomed) plate was contoured and applied to the medial surface of the femur and tibia, fixed with four screws in the femur and four screws in the tibia. A contoured eight-hole 2.7 mm SOP plate was then applied laterally to the surface of the femur and tibia, and fixed with three screws in the femur and two screws in the tibia. The tibial tuberosity was reduced and secured with two parallel 1 mm K-wires. The musculofascial incision, subcutaneous tissue and skin were all closed routinely.

**Figure 2:**
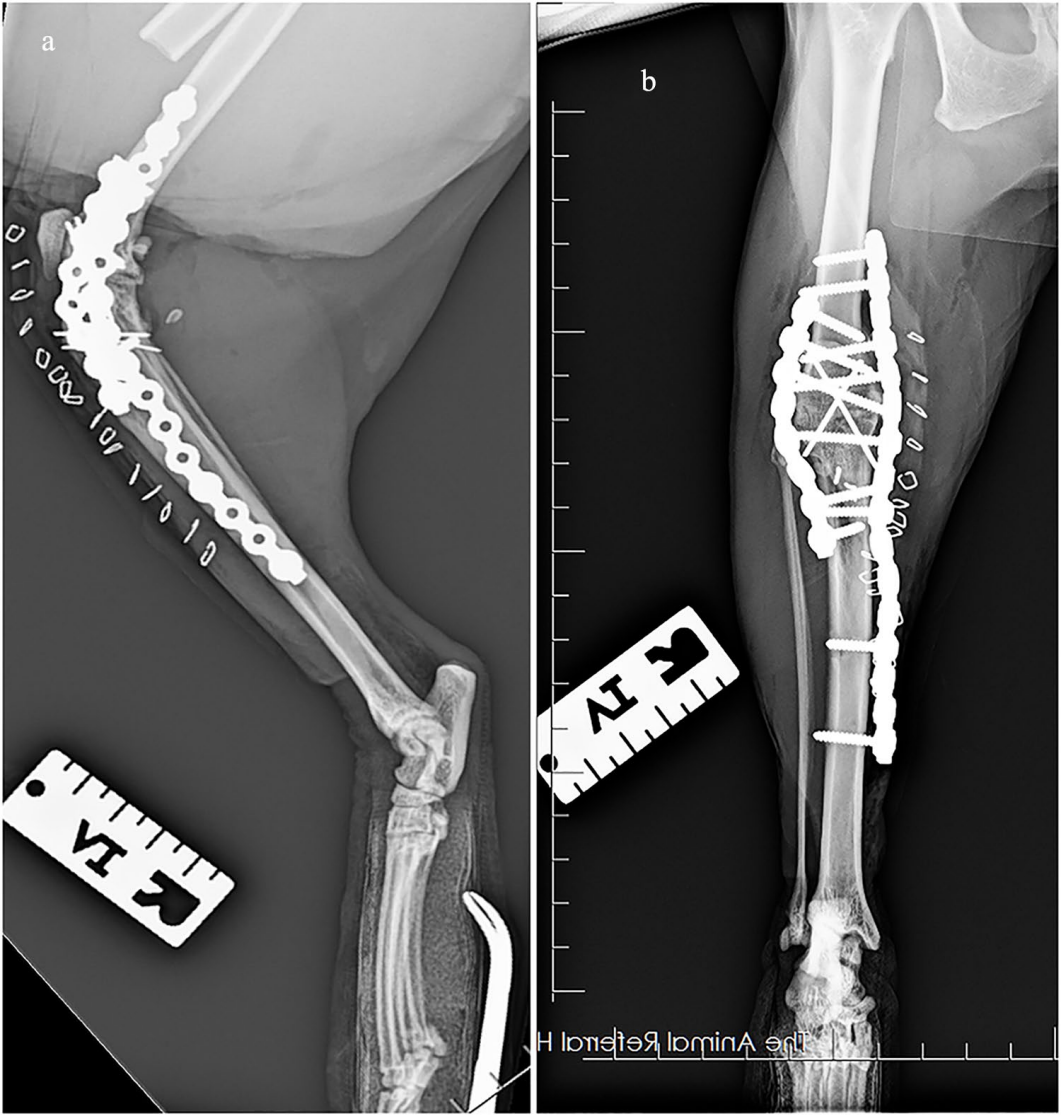
(a) Mediolateral radiograph of the right stifle immediately postoperatively. A 15-hole SOP plate has been applied medially and an eight-hole SOP plate has been applied laterally. Two 1.0 mm K-wires were placed in a parallel fashion to stabilise the tibial tuberosity osteotomy. (b) Craniocaudal radiograph of the right stifle immediately postoperatively. Two 1.4 mm K-wires were placed in a cross-pin fashion

Postoperative radiographs revealed satisfactory alignment, apposition and implant positioning (Figure 2). The stifle flexion angle was 110°. A soft padded bandage was placed postoperatively. The cat was maintained on a continuous rate infusion of fentanyl for intraoperative analgesia and cefazolin (22 mg/kg IV) was administered as perioperative antibiotic. Both medications were continued for 12 h postoperatively. The cat was then transitioned to methadone (0.2 mg/kg SC q6h) and oral meloxicam (0.05 mg/kg PO q24h).

The cat was hospitalised for another 7 days post-operatively due to the development of an abscess at the previous amputation site and a urinary tract infection. Under general anaesthesia, the abscess was drained, samples were submitted for culture and sensitivity testing, and a Jackson-Pratt drain was placed. The cat was then transferred back to the primary care veterinarian for continued management.

**Figure 3:**
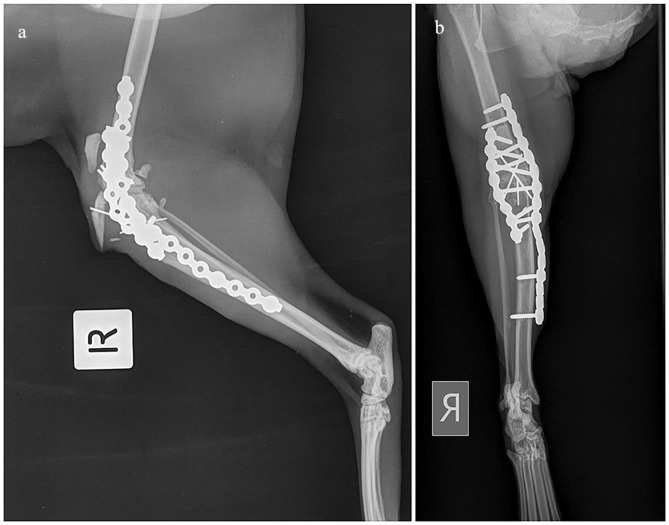
(a) Mediolateral radiograph of the right stifle 3 weeks postoperatively. Image shows displacement of the tibial tuberosity cranioproximally with one of the parallel K-wires. (b) Craniocaudal radiograph 3 weeks postoperatively

Two weeks postoperatively, the primary care veterinarian reported that the abscess had resolved and the cat was able to ambulate with sling assistance. Three weeks postoperatively, avulsion of the tibial tuberosity occurred (Figure 3). Revision surgery was performed to reduce the tibial tuberosity and secure it with three parallel 1.0 mm K-wires and a figure-of-eight tension band wire using 22 G orthopaedic wire. At 6 weeks postoperatively, the cat was able to rise and ambulate without support.

**Figure 4:**
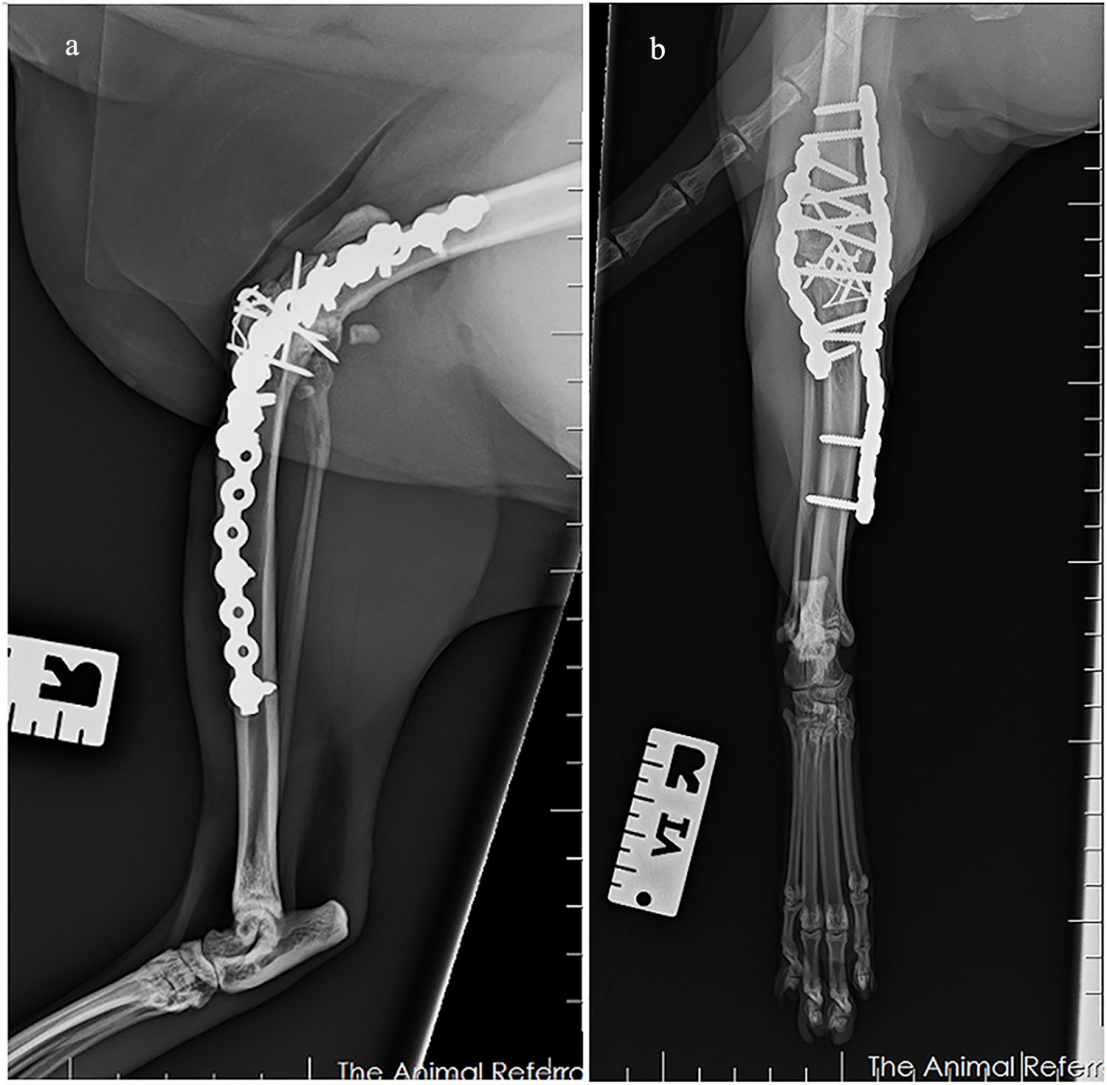
Five months postoperative (a) mediolateral radiograph and (b) craniocaudal radiograph

The cat returned to our hospital 5 months postoperatively for a recheck. On physical examination, no pain reaction was elicited on palpation over the implants. Although the cat had a mechanical lameness characterised by circumduction of the right pelvic limb, the cat was able to rise and walk without any apparent difficulties. Radiographs revealed stifle arthrodesis with no evidence of implant-related complications (Figure 4).

A phone update was obtained 1 year after the surgery. The owner reported that aside from an inability to squat during toileting, the cat had resumed a near-normal lifestyle.

The owner stated that she was satisfied with the outcome and believed that the cat had a good quality of life, justified on the basis of its similar activity level before the injury and after the surgery.

## Discussion

Primary joint reconstruction is desired whenever possible and has been reported to achieve good to excellent outcomes in the majority of cats.^4^ However, in this case, primary joint reconstruction was not possible due to the chronicity of the injury. Consequently, a stifle arthrodesis was deemed appropriate.

A prior case report has documented effective arthrodesis of the feline stifle joint with a single plate technique.^6^ As a previous canine biomechanical study has demonstrated increased load on the remaining pelvic limb after amputation, we chose to utilise a novel bilateral plate technique.^11^ Bilateral plating has been shown to increase axial, bending and torsional stiffness compared with unilateral plating.^12,13^ In addition, SOP plates instead of the previously reported dynamic compression plate were selected to stabilise the ostectomy. The beneficial features of the SOP plate include preservation of the periosteal blood supply, availability of variable lengths, the ability to contour the plate in six degrees of freedom and ease of contouring of the plate without drastically affecting its mechanical properties.^14^ In retrospect, a longer lateral and medial plate that extended over the proximal femur and the distal tibia, respectively, should have been used to reduce stress concentration within the bone diaphysis, thereby minimising the likelihood of postoperative fracture, as previously reported.^8,9^

The avulsion of the tibial tuberosity could have been avoided by placing a tension band wire at the initial surgery. The use of tension band wire has previously been shown to reduce complication rates as it converts tension force into compression force.^15^ In this case, we incorrectly theorised that the traction generated by the quadriceps mechanism would have been neutralised by the rigid immobilisation of the stifle joint.

The previous case report detailing feline stifle arthrodesis demonstrated successful joint fusion by debriding the articular cartilage of the distal femur and proximal tibia.^6^ In contrast, we decided to perform ostectomies to optimise load sharing and congruency between the distal femur and proximal tibia.^8^ Meticulous preoperative planning and execution of the ostectomies are required to achieve the desired joint angle and to avoid iatrogenically inducing an angular limb deformity. Although the postoperative stifle flexion angle of 110° was slightly smaller than the previously reported 120°, it does not appear to be clinically significant.^8^

This case report also highlights the importance of implementing a surgical checklist in veterinary practices. In human medicine, a reduction of postoperative complication rates by 36% has been documented, whereas in two veterinary studies, reductions of complication rates by 11.6% and 10% have been documented.^16–18^ A similar scenario has previously been reported in the veterinary literature where an incorrect limb had been draped and prepared for surgery.^19^ Fortunately, a preoperative checklist was performed that identified the mistake before a catastrophic error was made.^19^ It is highly likely that had a preoperative checklist been performed, the cat in this report would not have had the incorrect limb amputated.

In conclusion, this case report has demonstrated the tolerance of a stifle arthrodesis in an amputee cat.
